# Transtendon technique versus repair after completion of the tear for articular-sided partial rotator cuff tear: a meta-analysis of comparative studies

**DOI:** 10.1186/s13018-023-03831-4

**Published:** 2023-05-22

**Authors:** Yimeng Yang, Dan Hu, Shiyi Chen, Shibing Guan, Xiliang Shang

**Affiliations:** 1 Department of Sports Medicine, Huashan Hospital, No. 12, Wulumuqi Zhong Road, Shanghai, 200040 China; 2 State Key Laboratory of Molecular Engineering of Polymers, Fudan University, Shanghai, China; 3 Department of Orthopedics, The Affiliated Suzhou Hospital of Nanjing Medical University, Suzhou, 215008 China; 4 Department of Hand and Foot Surgery, Shandong Provincial Hospital Affiliated to Shandong First Medical University, Jinan, 250021 China

## Abstract

**Background:**

Transtendon repair and repair after completion of the tear have been widely used to treat partial-thickness rotator cuff tears (PT-RCTs). The present study was aimed to compare the clinical outcomes and tendon integrity following arthroscopic repair of articular PT-RCTs using transtendon repair or repair after completion of the tear.

**Methods:**

We performed a systematic electronic database search on Cochrane Central Register of Controlled Trials, PubMed and Embase to identify articles equating articular-sided PT-RCTs repair. The randomized controlled clinical trials that met our criteria were evaluated for quality of methodology. The results obtained were further analyzed and correlated to present the benefits and drawbacks comparing the two surgical procedures.

**Result:**

According to our inclusion and exclusion criteria, six articles were included in the present study. A total of 501 patients were analyzed as part of this study. The results indicated that both the surgical treatments provided excellent functional improvements and tendon integrity. However, no significant differences for the visual analogue scale (VAS) score, American Shoulder and Elbow Surgeons (ASES) score, constant score, range of motion, postoperative adhesive capsulitis, tendon integrity and patient satisfaction were found between the two cohorts (*p* > 0.05).

**Conclusions:**

Both transtendon technique and repair after completion of the tear for articular-sided partial rotator cuff tear provide improvements in clinical outcome with a low complication rate and a high rate of healing.

## Introduction

Partial rotator cuff tears (PT-RCTs) are a common cause of shoulder pain and disability and can be classified as bursal side, articular side, or intratendinous in accordance with the injury site [24]. Ellman developed a system based on the location and depth of the tear to effectively classify, diagnose, and treat PT-RCTs [7]. Most authors recommend repair of PT-RCTs involving > 50% of the tendon thickness (Ellman grade III) after failed conservative management, whereas PT-RCTs involving < 25% (Ellman grade I) are treated conservatively rather than surgically. However, the current evidence does not provide guidance as to the best arthroscopic treatment plan for symptomatic PT-RCTs [8, 9, 27, 28, 30]

Generally, surgical alternatives for treating symptomatic articular-sided PT-RCTs include arthroscopic repair following full-thickness conversion and various in situ repairs [18] Some surgeons prefer conversion from PT-RCTs into full thickness tears with remnant debridement, acquiring a better view and repair the remnant rotator cuff tissue using a familiar repair technique. This tear completion repair technique is reported to be satisfactory with good visualization, strain characteristics, and healing rates [18, 22]. However, the tear completion repair technique sacrifices the intact bursal-sided rotator cuff tendon and causes some complications due to damaged integrity of the tendon [19]. Therefore, some surgeons prefer the transtendon repair technique which restores the rotator cuff footprint while leaving the bursal side intact. Cadaveric biomechanical study reported that transtendon repair improve biological and biomechanical repair properties with less gap formation than the tear completion repair technique [12]. Nevertheless, some authors have raised concerns about the potential damage to the intact tendon during suture anchor insertion and overtension after the repair [22]. At the same time, this procedure is relatively complex with poor visualization for surgeons. Currently, tear completion repair and the in situ transtendon procedure are the two commonly adopted methods. However, the optimal surgery method in the treatment of articular-sided PT-RCTs remains controversial.

The purpose of this study was to compare functional outcomes and tendon integrity for patients with an articular-sided PT-RCTs who undergone arthroscopic transtendon repair or tear completion repair. We hypothesized that arthroscopic repair of articular-sided PT-RCTs would provide satisfactory functional improvements and tendon integrity regardless of the two repair technique.

## Materials and methods

### Search strategy

We carried out a meta-analysis of the literature with the search terms “partial”, “articular” and “rotator cuff tear”. A complete search of the literature in the following databases was performed: MEDLINE (PubMed) (1950 to June 2021), Embase (Ovid) (1974 to June 2021) and Cochrane (1996 to June 2021). Our inclusion criteria included outcome-based studies of isolated partial articular-sided rotator cuff tears (> 50% of the thickness of the tendon) which compared arthroscopic transtendon repair and tear completion repair by using clinical or functional scoring systems. Exclusion criteria included studies that involved cadaver or animal studies, biomechanical studies, literature reviews, letters to editors, expert opinion articles, case reports or technique notes which did not contain clinical outcome-based data.

### Quality assessment

The Coleman methodology score (CMS) was applied to determine the quality of the involved studies according to our previous study [21]. The CMS consists of 15 items in its checklist and is scaled from 0 to 100 points. A score from 85 to 100 is considered excellent, 70–84 as good, 55–69 as fair and below 55 as poor. An overall score of 100 suggests that the study avoids chance, bias and confounding variables. The quality assessment by CMS was carried out by two independent reviewers. In addition, all the results were confirmed by the senior author.

### Outcome measures

The identified studies were measured and analyzed for the following outcomes: visual analogue scale (VAS) score, American Shoulder and Elbow Surgeons (ASES) score, Constant score, range of motion, postoperative adhesive capsulitis, tendon integrity and patient satisfaction. Two reviewers evaluated the literature separately, and any discrepancies were reevaluated and resolved by consensus.

### Statistical analysis

A formal meta-analysis was conducted only for clinical outcome data from comparative studies using Stata 12.0 (Stata Corp LP, College Station, TX, USA). Results for continuous or categorical outcomes were reported as a mean difference or an odds ratio, respectively, with 95% confidence intervals.

## Results

### Literature search

**Fig. 1:**
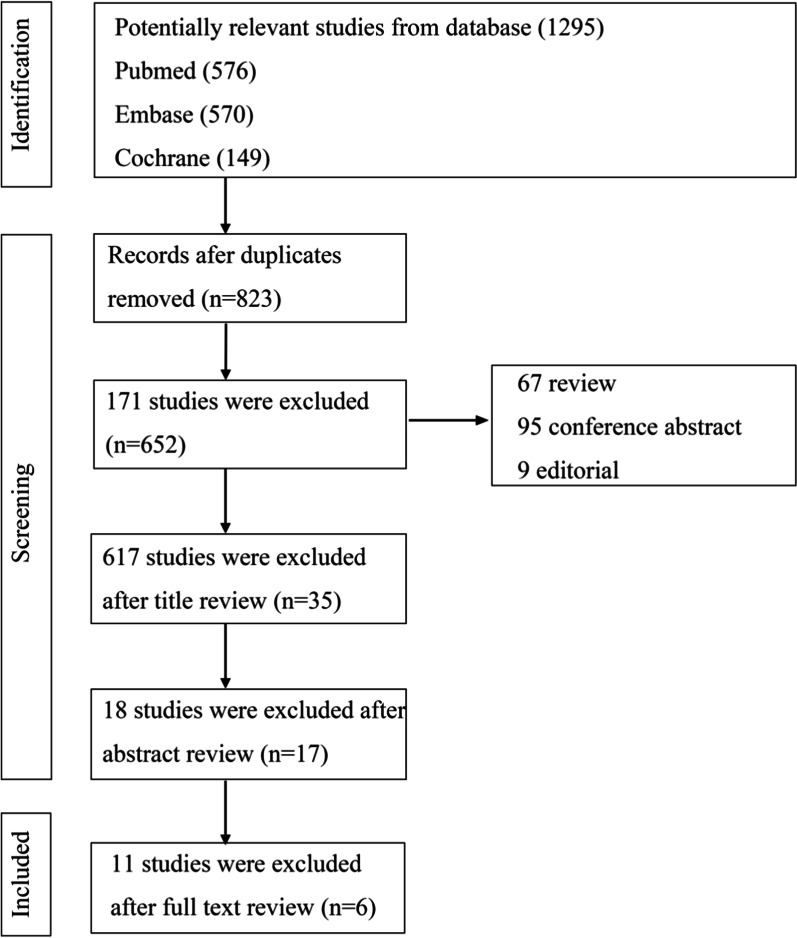
Search strategy flow diagram

A total of 1295 studies were selected after an intensive database search on PubMed (*n* = 576), Embase (*n* = 570) and Cochrane (*n* = 149). Six articles that met our inclusion and exclusion criteria were subsequently included after a full-text review (Fig. 1). Four were prospective comparative study and two were retrospective comparative study (Table 1).

**Table 1:** Characteristics of the included studies

Author	Study, LoE	Participants	Intervention	Outcomes
Shin et al. [29]	Prospective comparative study, II	48 patients who underwent arthroscopic repair of articular-sided PT-RCTs between March 2006 and July 2008 were included	24 transtendon repair 24 repair after completion of the tear	VAS score; ASES score; Constant score; Range of Motion; Patient satisfaction; MRI features
Franceschi et al. [10]	Prospective comparative study, II	60 patients who underwent arthroscopic repair of articular-sided PT-RCTs from 2007 to 2009	32 transtendon repair 28 repair after completion of the tear	ASES score; Constant score; Range of Motion; MRI features
Kim et al. [19]	Prospective comparative study, II	92 cases between January 2008 and March 2011 with articular-sided PT-RCTs exceeding 50% of tendon thickness	47 in Situ Repair 45 Tear Completion Repair	VAS score; ASES score; Constant score; MRI features
Castagna et al. [2]	Prospective comparative study, II	74 patients between 2006 and 2009 underwent an arthroscopic rotator cuff repair of articular-sided PT-RCTs	37 transtendon repair 37 repair after completion of the tear	VAS score; Constant score;
Liu et al. [22]	Retrospective comparative study, II	68 patients between December 2014 and June 2015 underwent rotator cuff repair	30 transtendon repair 38 repair after completion of the tear	VAS score; ASES score;
Castricini et al. [5]	Retrospective comparative study, II	151 patients (153 shoulders) who underwent rotator cuff repair between January 2003 to December 2014	59 transtendon repair 94 repair after completion of the tear	Constant score; simple shoulder test (SST); Tendon integrity (Ultrasound); Patient satisfaction

### Surgical outcome score

**Fig. 2:**
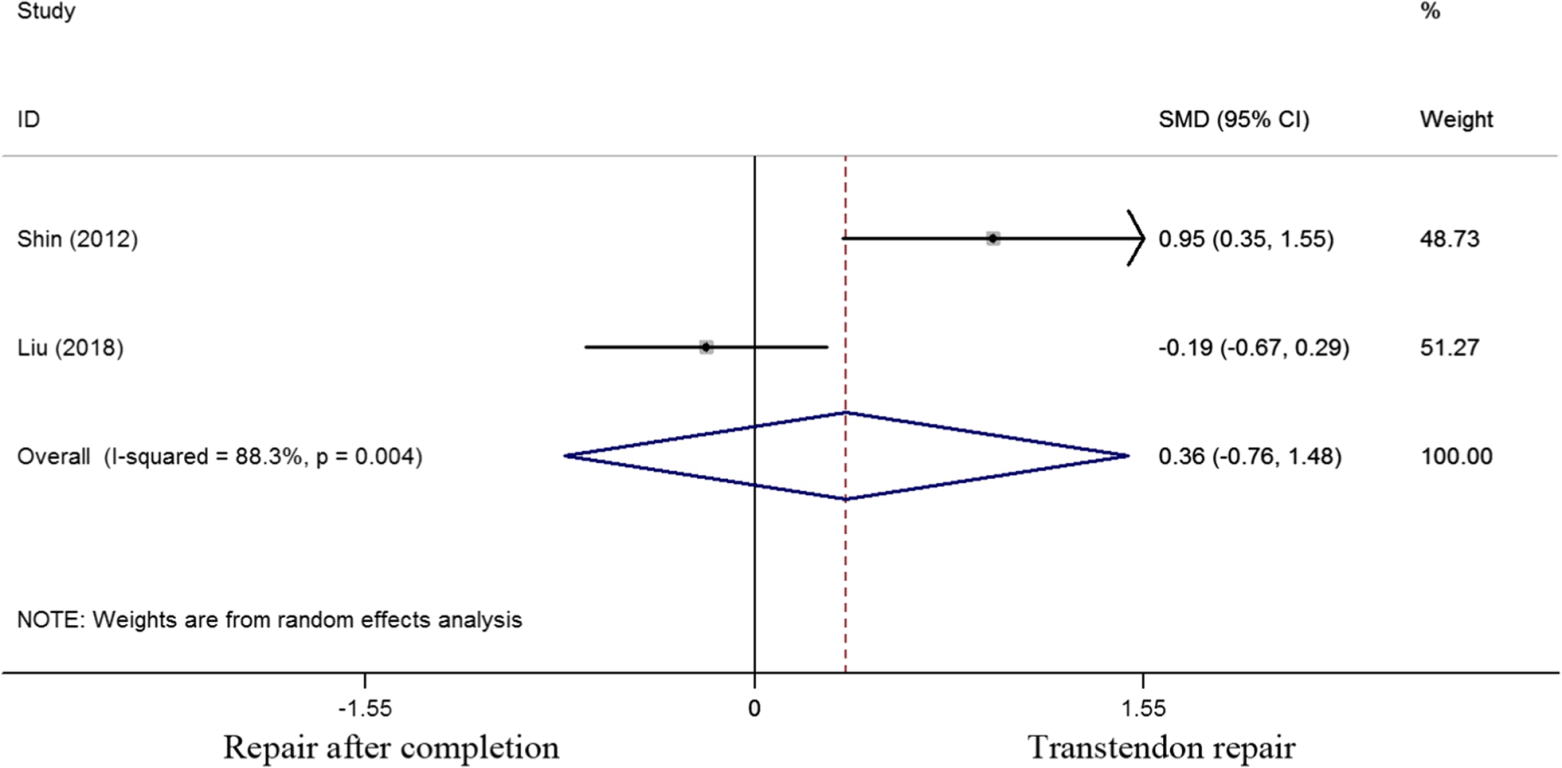
Standard differences in means for VAS score between transtendon repair and repair after completion of the tear groups

Patients were clinically assessed both preoperatively and postoperatively on a number of outcome-based scores that included the VAS score, ASES score, Constant score, range of motion, postoperative adhesive capsulitis, tendon integrity and patient satisfaction. The VAS score was evaluated in two studies. And the standard mean difference of VAS score was 0.36 (-0.76 to 1.48). No significant difference was observed regarding the VAS score between the two cohorts (P = 0.525) (Fig. 2).

**Fig. 3:**
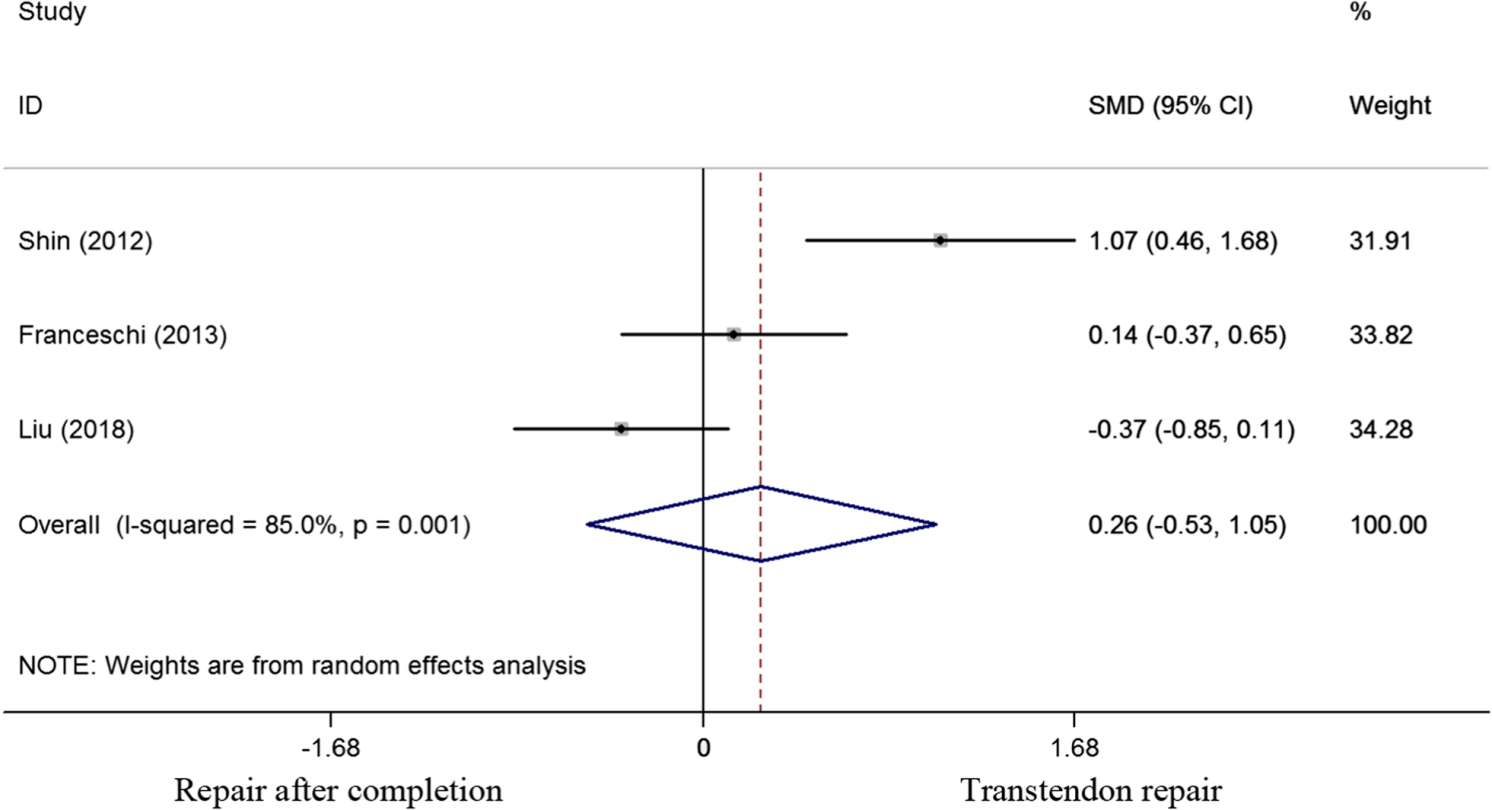
Standard differences in means for ASES score between transtendon repair and repair after completion of the tear groups

ASES score was examined in three studies. The standard mean difference of ASES score was 0.26 (-0.53 to 1.05). No significant difference was observed between the two cohorts for the ASES score (P = 0.515) (Fig. 3).

**Fig. 4:**
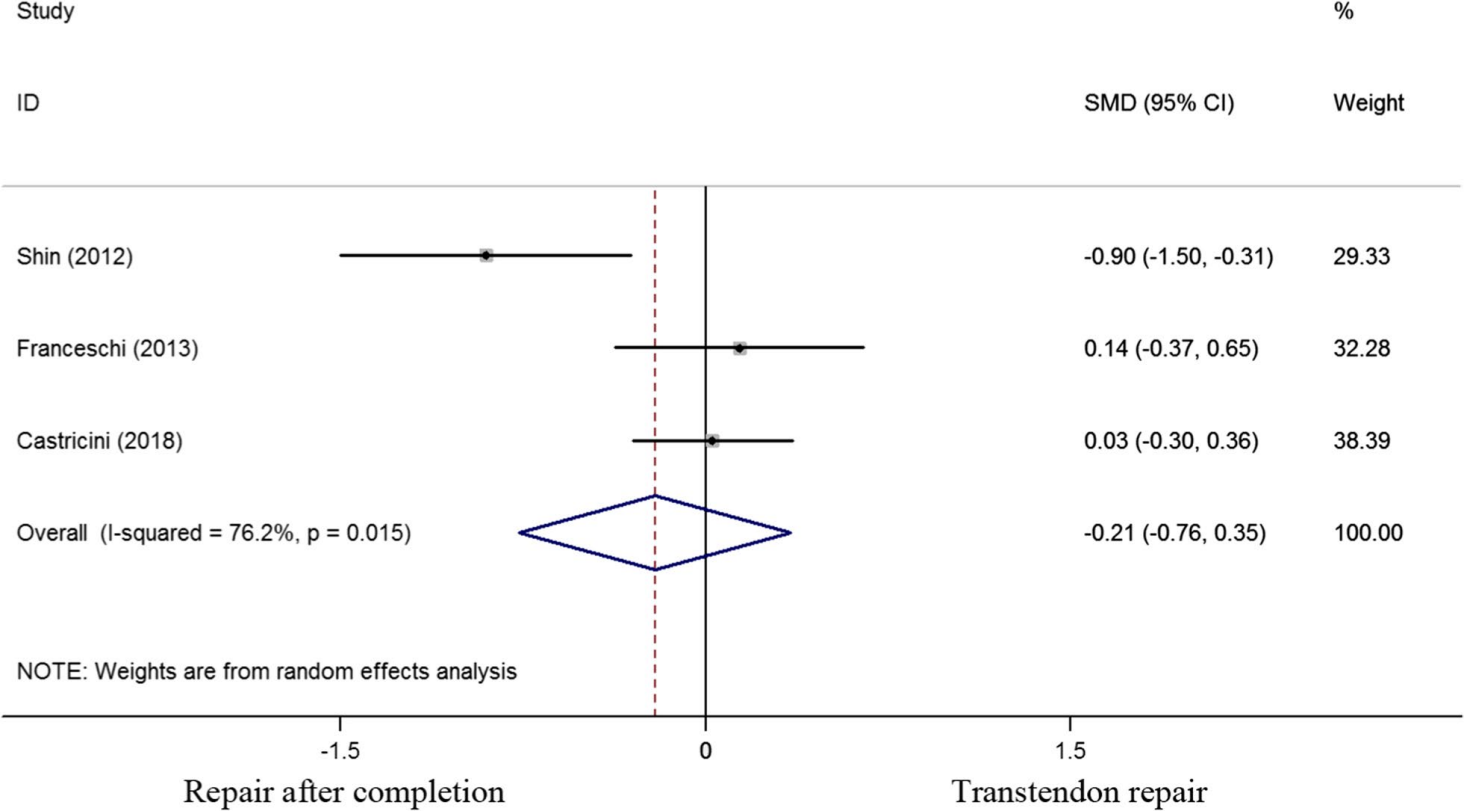
Standard differences in means for constant score between transtendon repair and repair after completion of the tear groups

The constant score was evaluated in 3 of the 6 studies. The standard mean difference of constant score was 0.21(-0.76 to 0.35), implying that no significant difference was found between the two cohorts (P = 0.463) (Fig. 4).

**Fig. 5:**
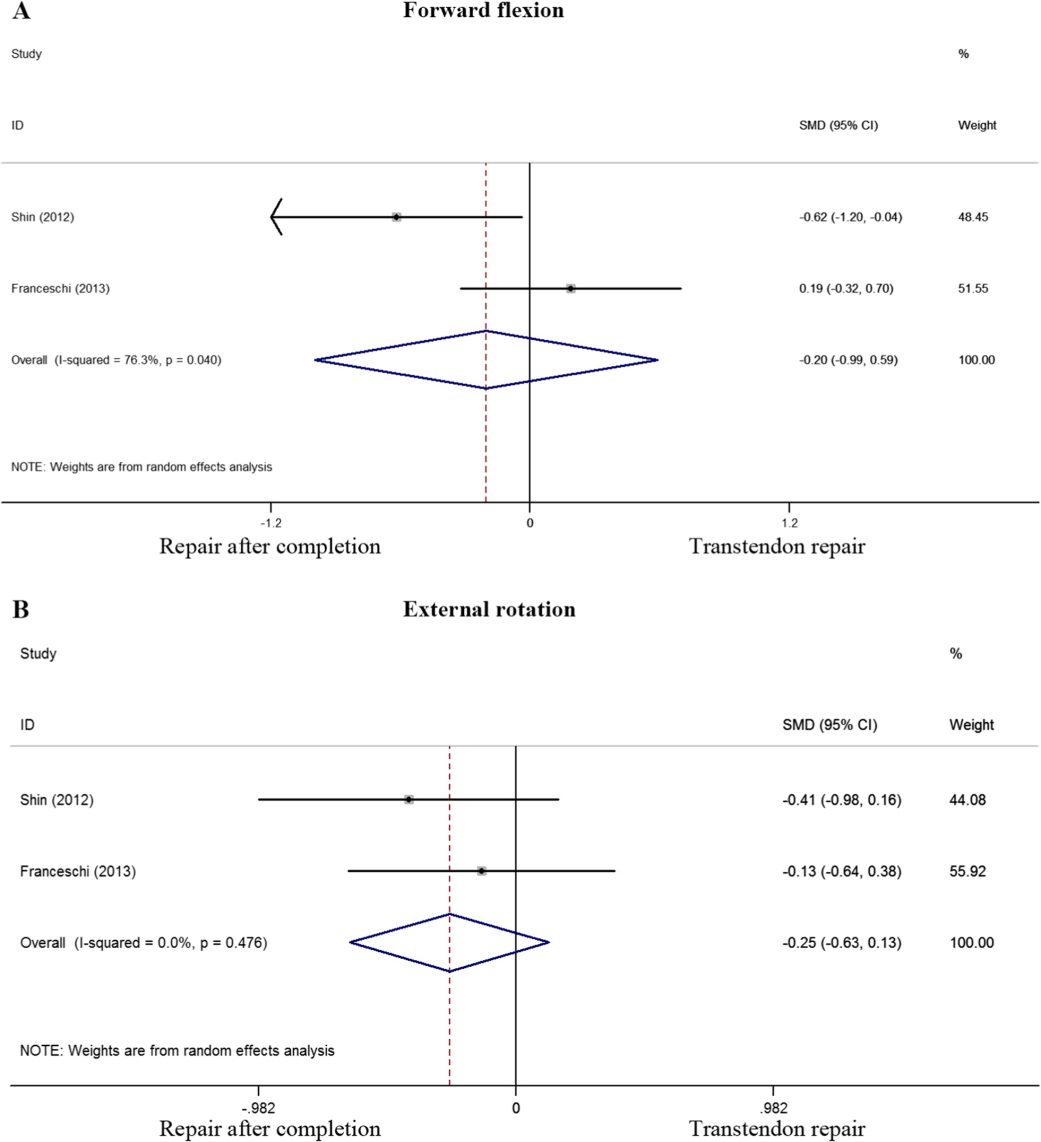
Standard differences in means for forward flexion (A) and external rotation (B) between transtendon repair and repair after completion of the tear groups

**Fig. 6:**
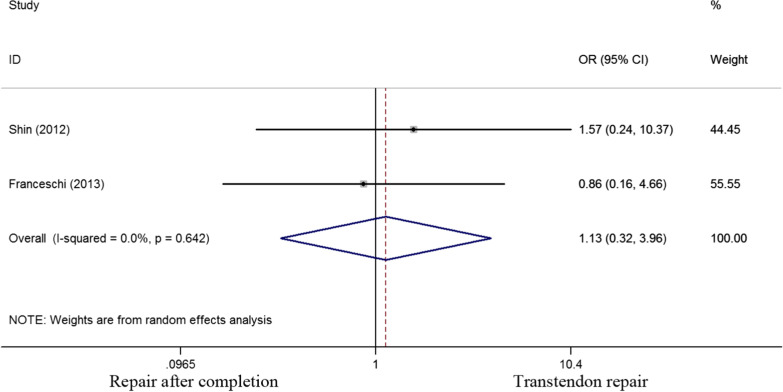
Odds ratios for postoperative stiffness between transtendon repair and repair after completion of the tear groups

The range of motion was evaluated in 2 of the 6 studies. In terms of forward flexion, a standard mean difference of -0.203 was determined (P = 0.615) while the standard mean difference of external rotation was -0.254 (P = 0.190). No significant difference was found between the two cohorts (*p* > 0.05) (Fig. 5). Two of six studies selected had evaluated postoperative stiffness. An odds ratio of 1.13 (0.32 to 3.959) was calculated with a P value of 0.854, implying no significant difference in postoperative adhesive capsulitis between the two groups (Fig. 6).

**Fig. 7:**
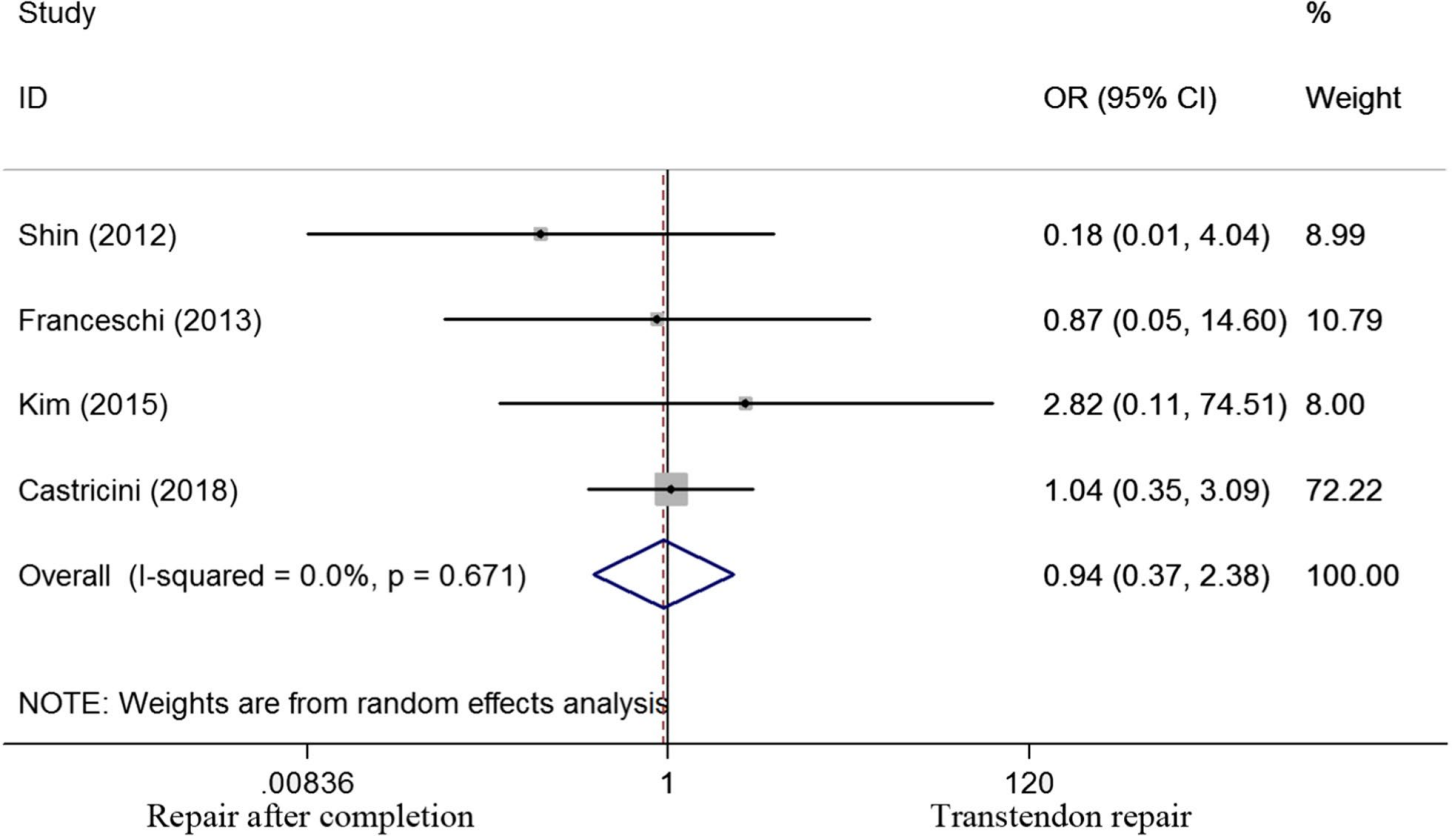
Odds ratios for tendon integrity between transtendon repair and repair after completion of the tear groups

**Fig. 8:**
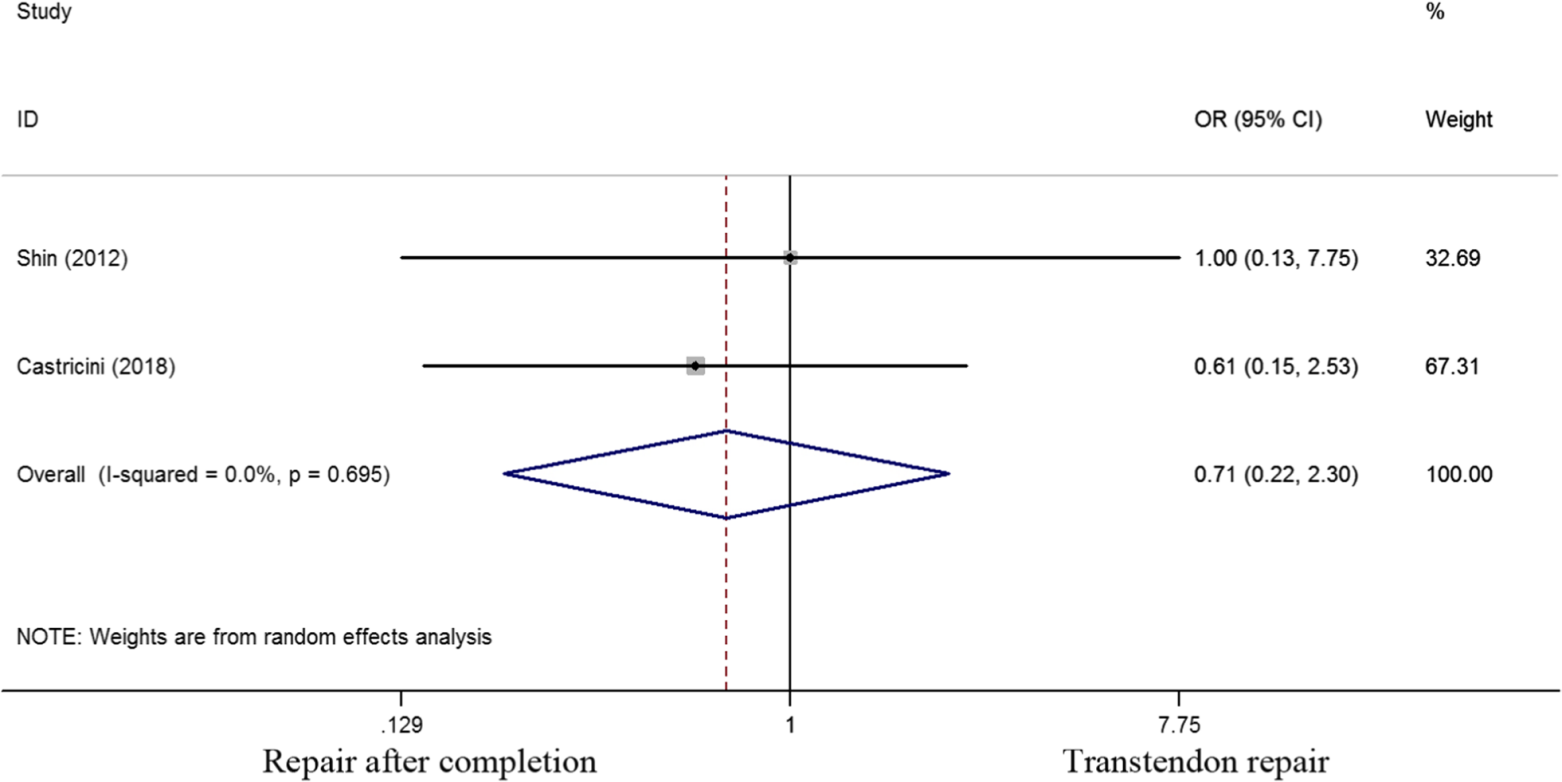
Odds ratios for patients satisfaction between transtendon repair and repair after completion of the tear groups

Four of six studies selected had evaluated tendon integrity. An odds ratio of 0.944 (0.374 to 2.384) was calculated with a P value of 0.903, implying no significant difference in tendon integrity between the two groups (Fig. 7). Two studies evaluated the patients satisfaction outcome. An odds ratio of 0.714 (0.222 to 2.303) was found with a P value of 0.573, thereby indicating that no significant difference in patients’ satisfaction between the two groups (Fig. 8).

## Discussion

The main finding of our study is that both procedures provide improvements in clinical outcome with a low complication rate and a high rate of healing. No significant difference in clinical outcome was demonstrated between these two procedures for articular-sided partial rotator cuff tear repair.

Articular PT-RCTs with a thickness of greater than 50% can be repaired by tear completion repair and the in situ transtendon procedure. Tear completion repair is a relatively straightforward method for restoration of the tendon footprint with satisfactory clinical outcomes and high levels of patient satisfaction. Some authors advocated tear completion repair because it allowed better access to the tendon footprint and tended to be more convenient to secure tendon fixation [6]. Furthermore, after the removal of degenerative tissue, it could obtain an advantageous healing microenvironment that is akin to an acute full-thickness tear [1, 17]. Gereli et al. found the completion repair technique exhibited increased healing characteristics compared with the in situ technique in spite of the concerns of detaching the intact tendon [11]. The reason for this finding might be the refreshing effect of debridement at the chronic degenerated tendon that could improve the healing response [11]. Although this technique achieved good clinical outcomes, it cannot to anatomically repair the lesion and restore native length–tension match after surgery [10, 15]. Therefore, some authors raised its concerns of biological and mechanical flaws, which could reduce tendon integrity potential and result in functional disabilities overtime.

In contrast, the transtendon repair made it possible to better restore the rotator cuff footprint anatomically and maintain the tendon integrity as preserving the bursal side rotator cuff tendon [1]. Cadaveric study has demonstrated that transtendon repair technique creates smaller gap formation and higher ultimate tensile loads than tear completion repair technique [12, 25]. Meanwhile, some studies have found that transtendon repair is an effective treatment for articular PT-RCTs with significantly improved functional scores and pain relief [3, 10, 26, 29]. Sun et al. conducted a meta-analysis to compare the two techniques for treating articular-sided PT-RCTs of more than 50% thickness [31]. They found that the trans-tendon technique is better than the tear conversion followed by repair technique with regard to the management of articular-sided PTRCTs of more than 50% thickness in the re-tear rate aspect [31]. Despite complete integrity, slower shoulder functional improvements during the recovery period and higher occurrence rate of postoperative shoulder stiffness after transtendon repair have been reported [13, 16, 23]. Jordan R W et al. reviewed the incidence of post-operative stiffness of articular PT-RCTs using transtendon repair or tear completion repair [16]. The included case series demonstrated a higher rate of stiffness in the transtendon repair group (range 0 to 18% compared to 0 to 2.8% after completion and repair). Furthermore, the shoulder discomfort after the transtendon repair technique might was caused by unbalance the tension of the remaining torn cuff because of a retracted articular rotator cuff layer and overtightening the bursal portion of the cuff [4, 14, 29]. This altered tensioning on both sides of rotator cuff tendons may be the cause of the shoulder stiffness. Although various modifications of transtendon repair techniques have been introduced to avoid overconstraining the joint and tendon overstrain, improved arthroscopic instruments and transtendon repair techniques are needed to reduce risks of postoperative morbidity [29]. On the other hand, the remaining cuff tissue has already showed histopathological degeneration and would be a painful nidus causing early postoperative pain [11, 32]. Yamakado, Kotaro has taken biopsy specimens of the residual tendon in 30 consecutive patients with articular side PT-RCTs [32]. Samples were histopathologically examined and graded by use of a modified semiquantitative scale. The study showed that degenerative changes were evident in 28 of 30 cases (93%) and over 90% of the macroscopically intact residual tendon showed moderate histopathologic degeneration. But no difference was found between the two repair techniques with respect to VAS score in the present study. Further studies are required to determine the effect of remaining cuff tissue on early postoperative recovery and long-term clinical outcome. These findings together suggest that the biomechanical advantage of preserving the tendon integrity with transtendon repair does not provide better functional results and healing rates compared with tear completion repair.

The limitations of this study include a relatively small number of studies. Despite the small sample size, we included only level- II studies. Hence, these studies represent high-level evidence on a large number of patients. The preliminary results presented in this study would help to clarify clinical outcomes of both repair techniques. In addition, the heterogeneity of imaging modality may represent a weakness of the study. There is a lack of standardized evaluation method for accurate assessment of tendon thickness. According to a previous report, ultrasound and MRI have good and similar diagnostic accuracy for detection of rotator cuff tears, and they have been widely performed to evaluate rotator cuff integrity [20].

## Conclusions

Arthroscopic repair of partial-thickness articular-sided rotator cuff tears provided functional improvements and tendon integrity regardless of the transtendon repair and repair after completion of the tear. Due to the simple operation and the removal of an obvious degeneration of the rotator cuff, the procedure of repair after completion of the tear is proposed in the case of obvious degeneration of rotator cuff.

## Data Availability

All data generated or analyzed during this study are included in this article. The data are available from the corresponding author upon reasonable request.
